# Processing of Scalar Inferences by Mandarin Learners of English: An Online Measure

**DOI:** 10.1371/journal.pone.0145494

**Published:** 2016-01-11

**Authors:** Yowyu Lin

**Affiliations:** Department of Foreign Languages and Literatures, National Taiwan University, Taipei, Taiwan; University of Akron, UNITED STATES

## Abstract

Scalar inferences represent the condition when a speaker uses a weaker expression such as *some* in a pragmatic scale like <some, all>, and s/he has the intention to reject the stronger use of the other word like *all* in the utterance. Considerable disagreement has arisen concerning how interlocutors derive the inferences. The study presented here tries to address this issue by examining online scalar inferences among Mandarin learners of English. To date, Default Inference and Relevance Theory have made different predictions regarding how people process scalar inferences. Findings from recently emerging first language studies did not fully resolved the debate but led to even more heated debates. The current three online psycholinguistic experiments reported here tried to address the processing of scalar inferences from second language perspective. Results showed that Mandarin learners of English showed faster reaction times and a higher acceptance rate when interpreting *some* as *some but not all* and this was true even when subjects were under time pressure, which was manifested in Experiment 2. Overall, the results of the experiments supported Default Theory. In addition, Experiment 3 also found that working memory capacity plays a critical role during scalar inference processing. High span readers were faster in accepting the *some but not all* interpretation than low span readers. However, compared with low span readers, high span readers were more likely to accept the *some and possibly all* condition, possibly due to their working memory capacity to generate scenarios to fit the interpretation.

## Introduction

Successful second language comprehension and production involve building grammatical structures rapidly from incoming linguistic input and then producing appropriate responses. Within milliseconds, interlocutors need to decode incoming phonemic and morphological strings, construct syntactic representations, integrate semantic information and finally respond appropriately to their interlocutors. Sentence comprehension, however, is not always easy and smooth. Sometimes if utterances involve complex grammatical structures, comprehenders need to spend more time analyzing these structures. Sometimes, when the meaning of utterances is not straightforward and involves additional meaning, comprehenders need to be capable of being aware of it so that they can “read between the lines.” These seemingly daunting and complex tasks are nevertheless conducted in an extremely rapid manner for first language speakers, who rarely encounter any difficulty during sentence comprehension. However, any of these tasks could potentially be a huge challenge for second language learners and could lead to comprehension or production breakdown. Many studies have shown that adult second language speakers, although equipped with superior linguistic knowledge and proficient second language skills, still display non-native-like proficiencies in many subtle aspects. For example, the English article system poses a difficult challenge even for advanced English learners [1, 2, 3, 4]. In addition, many different theories have been proposed to account for the fact that second language learners generally fail to attain a native-like proficiency level [5, 6, 7].

In recent years, advances in computers and imaging technology have provided the driving force for online examination and understanding of the cognitive mechanism of second language learners. Such cutting-edge technology also provides researchers the ability to examine the validity of different theories. These online techniques include reaction times, event-related potential (EPRs), functional Magnetic Resonance Imaging (fMRI), transcranial magnetic stimulation (TMS) and positron emission tomography (PET) [8, 9, 10, 11]. The advancement of modern techniques has enabled researchers to form research questions about first and second language acquisition and comprehension that could not be answered previously. For example, researchers have been using fMRI to see whether the neural substrates are the same between first and second language speakers and how other factors such as age and proficiency could influence cerebral organization among second language learners. Despite the discrepancies found among different studies using different techniques [8, 9, 12], Perani et al. [13] did find that proficiency level and age in a second language play an important role in second language processing.

Most of the studies investigating second language learning have focused on syntax [14], phonology [15], morphology [16], pedagogy [17] and so on. Few have looked at second language learning or processing in pragmatics using online methods to address these issues. The current paper attempts to bridge this gap by using the online reaction time technique to examine scalar inferences in second language learners. To be more specific, the author investigated Mandarin learners’ processes of English scalar inferences. Before I delve into our research questions and design, I will first briefly introduce Grice’s Cooperative Principles and scalar inferences in pragmatics.

## Theoretical Background

From a linguistic point of view, human communication involves decoding and encoding incoming and outgoing strings of utterances. However, language communication conveys much more than what is encoded superficially. For example, when one interlocutor says, “Some of my sons are doctors,” one of the possible assumptions that is not encoded in this utterance is “Not all of my sons are doctors.” Thus during a conversation, a listener has to go beyond the literal linguistic expressions, or read between the lines, to get the implicit meaning, or *implicatures*, from the speaker. The processes of deriving these inferences among interlocutors were first characterized by a philosopher of language, Paul Grice [18], who systematically studied the meanings and inferences between interlocutors and laid the foundations of the modern study of pragmatics. He assumed that a successful communication involves cooperation between interlocutors and thus proposed the *Cooperative Principle* and four maxims to illustrate how people follow these rules to make conversation go smoothly.

***Cooperative Principle***: Contribute what is required by the accepted purpose of the conversation.
**Maxim of Quality**: Make your contribution true; do not convey what you believe to be false or unjustified.
**Maxim of Quantity**: Be as informative as required.
**Maxim of Relation**: Be relevant.
**Maxim of Manner**: Be perspicuous; avoid obscurity and ambiguity, and strive for brevity and order.

A typical implicature can be found in the following short conversation:

(1)**Speak A:** Did you do your laundry?
**Speaker B:** I was busy

In this conversation, it is quite obvious that B didn’t address A’s question directly. On the contrary, B replied with “I was busy” to imply that he did not do his laundry. According to Grice’s Cooperative Principle, if B is being cooperative, his contribution, or implicature, would be helpful in aiding his interlocutor to understand that he did not do the laundry. Grice has characterized the implicature like the example above as a *particularized implicature* since it occurs out of specific context, instead of arising from the linguistic or literal meaning of the words in the conversation. The latter one is known as a *generalized implicature*, which does not depend on a particular context to derive the meaning and can be illustrated by the following classic example:

(2) **a**. He is an Englishman; he is, therefore, brave.
**b**. His being an Englishman implies that he is brave.

In example (2), when (2a) is expressed, it entails (2b). This implication does not arise out of a specific context, as in (1), and is purely based on the linguistic or literal meaning of the words. In addition to context that distinguishes conversational implicatures from conventional implicatures, two other features also characterize conversational implicatures: *cancellable* and *reinforceable*. For example, what B said in (1) can be interpreted or inferred differently under a totally different context. In other words, the implicature that *B didn’t do the laundry* would be cancelled without the context of A’s utterance. These two features do not hold for conventional implicatures since whenever (2a) is expressed, it will always entail (2b).

## Scalar Inferences

Another form of conversational implicatures concerns scalar inferences [19, 20, 21], which involve using quantifiers like *all* or *some* in conversation. Horn [19] stated that logical words like <or, and> or quantifiers like <some, all> actually form information scales. They form a scale because statements such as “A and B” are subsets of statements like “A or B.” Thus the logical words can be ordered in the subset/superset order. This subset/superset relationship can be applied to quantifiers such as <some, all>, with *some* being the lower ranking term and *all* being the higher ranking term. For example, assuming that a speaker complies with Grice’s maxims and says as much as s/he can and says what is relevant to the conversation, when s/he says, “*I ate some of the cake*,*”* it is possible that the speaker implicates "I did not eat all of the cake," using *some* as a less informative or a lower ranking term than *all*. The main idea behind scalar inferences is that the use of a lower ranking term by the speaker prompts the hearer to realize the speaker’s reluctance to use the higher ranking one, i.e., *all*. In the conversation when the speaker chooses to select “some,” it is possible that the speaker rejects the use of the higher-ranking and stronger sense “all,” which might logically be not true or not warranted. <some, all> is not the only set of scalar inferences. Other possibilities include the use of numerals, modals, adverbs, lexical contrast and partial contextual orderings. Here are some examples of them.

(3) Numerals:
**John:** Do you have three chairs?
**Mary:** I have two.
**Implicature:** Mary does not have three chairs.
(4) Lexical contrast:
**Bryan:** Did you succeed in getting on the tennis team?
**Judie:** I tried.
**Implicature:** Judie did not succeed in getting on the tennis team.
(5) Partial Contextual Ordering
**Daniel:** Did you go to work today?
**Jennifer:** I went shopping.
**Implicature:** Jennifer did not go to work.

Utterances like “some X are Y” can be further illustrated by two inferences: “some and possibly all” and “some but not all.” These two interpretations of “some” can also be viewed as either being encoded logically or pragmatically. Logically speaking, “some” can be interpreted as “some and possibly all,” and is encoded by semantic rules. Pragmatically speaking, “some” is interpreted as “some but not all,” as required by real-world scenarios. Note that logically, there are four possibilities to account for “some X are Y.” These four possibilities include “X is a subset of Y,” “Y is a subset of X,” “X overlaps with Y” and finally “X coincides with Y.” The interpretation “some and possibly all” is considered less informative since it is compatible with all four possibilities. Compared with “some and possibly all,” “some but not all” reduces the four possibilities to only two possibilities and thus “some but not all” is considered to be more informative.

The derivation of implicatures in the original Gricean account has been described as complex and onerous since Grice originally considered that in addition to what has been said, interlocutors also have to evaluate what has not been said in a context. However, it does not seem plausible and efficient from cognitive development and sentence processing stages. If the implicature derivations are too costly, they would not benefit human cognitive development or mental processing. Since the Gricean theory was introduced, two influential accounts have been proposed to explain scalar inferences: the Default Inference and Relevance Theory. In Default Inference, Neo-Gricean researchers [20, 21, 22, 23, 24, 25] like Levinson [20] pointed out scalar inferences resulting from Q-heuristic, which states that “What isn’t said isn’t the case.” When a speaker uses a weaker form, s/he intends *not* to use the stronger form since the stronger form is not warranted or called for. This has been considered the default inference among interlocutors. Under this assumption, scalar inferences belong to generalized conversational implicatures, which should be derived automatically without considering the context and can speed up the cognitive processes. For Levinson, since the generation of scalar inferences is automatic or by default, it can only be cancelled when the context requires it. In the case of “some but not all” versus “some and possibly all”, the Default Inference predicts that interlocuters would automatically assume the “some but not all” interpretation, which should be least onerous during cognitive computation.

On the other hand, pragmatists who are in favor of contextual accounts are against the default view. According to them, a listener either narrows down or broadens the speaker’s linguistic expressions. Consider the example of “some X are Y” in the above cases. Interpreting *some* as “some and possibly all” is an example of broadening the scope of *some* while interpreting *some* as “some but not all” is an example of narrowing down its meaning. If a listener does not find a scenario that matches the use of *some and possibly all*, he would realize a narrowing use of *some* must be applied instead. Furthermore, some pragmatists argue that inferences that the listeners draw depend on how relevant the inferences are. For example, Relevance Theory, which offers a comprehensive account on inferences [26], postulates that when hearing an utterance containing a weaker form like *some*, the interpretation “some and possibly all” suffices since a less informative interpretation might be enough for listeners rather than “Some but not all,” which is more informative and requires listeners to apply Q-heuristic. Unless the speaker wants the listener to apply Q-heuristic, more cognitive effort is associated with generating a more informative “some but not all” interpretation. In this way, Relevance theory predicts the opposite from the Default Inference theory in the scalar inferences. In the example of “some,” the Relevance theory assumes that the listener would tend to go with the logical interpretation. In other words, when hearing “some X are Y”, interlocutors tend to interpret it as “some and possibly all.” If there is a need for the listener to go further, interlocutors would adopt the “some but not all” pragmatic interpretation.

## Previous Second Language Studies

Previously, pragmatics has relied heavily on theorists’ knowledge in theoretical linguistics and intuitions about the language. Experiments on scalar inferences in either first language or second language studies have not relied on experimental techniques until very recently. Studies on first language scalar inferences have begun to use various online techniques to investigate this issue [27, 28, 29, 30]. As shown in the previous sections, Gricean-, Neo-Gricean or Relevance theories have been trying to offer their accounts of pragmatics but none of them has been very explicit about the derivational process in the human cognitive system. In addition, despite many efforts trying to unravel implicatures, surprisingly few studies have been devoted to the psychological reality of scalar inferences. In two of the earlier studies, Bouton [31, 32] compared native speakers’ and non-native speakers’ interpretation of implicature in an attempt to investigate whether it was necessary to teach implicature to second language learners. Bouton found that L2 learners of English comprehended the implicature only 75% of the time. In a follow-up study, Bouton (1992) [32] used different methods, including structure, cloze, dictation, etc., to examine thirty L2 English learners’ comprehension of implicature and again found that even though his L2 subjects made improvement on scalar interpretations over time, their interpretation performances were still significantly different than those of native speakers.

Several downsides could be found in Bouton’s study. First, the study was a continuation of an even earlier study and given that the author decided to use the same group of subjects, it turned out that not many subjects wanted to take the same test again. The loss of many of the original subjects in the second study made the comparison between the two studies difficult. Second, Bouton did not explain the rationale for using four different methods in his study and did not describe whether there was a significant difference among them. Whether each method yielded the same pattern of implicature comprehension could not be compared across four methods. Third, Bouton’s conclusion was mainly drawn from looking at the results in proportions among groups of subjects. More adequate statistical analysis and interpretation would have been needed before any conclusions could be drawn are expected before making any conclusions.

In a more recent study examining French continuous and discontinuous quantifiers, Dekydtspotter and Hathorn [33] used the Truth Value Judgment Task to investigate whether English learners of French could correctly observe the subtle differences of a target sentence like “Something remarkable was observed by each of the researchers.” A continuous or a discontinuous French quantifier leads to subtle differences in interpretations. For a continuous quantifier, the interpretation can be either “all the researchers observed a different remarkable object” or “all the researchers observed the same object.” For a discontinuous quantifier, the interpretation can only be “the researchers did not observe the same object.” Dekydtspotter and Hathorn found that language learners with high language proficiency displayed native-like implicature interpretation while low-intermediate language learners did not show such a tendency. In a study investigating the use of contexts in deriving scalar implicatures among English and Korean first and second language speakers, Slabakova [34] found that Korean learners of English used more pragmatic interpretation in their second language than in their native language, with or without the context, and concluded that pragmatic interpretations were of no problem to second language learners. Given that no beginning learners of either English or Korean were involved in this study, it is unknown whether the same conclusion can be drawn for beginning learners.

Considerable disagreement has arisen concerning the cognitive mechanism underlying the processing of scalar inferences. In addition, how second language learners process scalar inferences is not fully understood yet. How do second language learners’ performances differ from those of native speakers? Would they be influenced by the same factors that affect L2 scalar inference processing? Or would they be less influenced by these L2 factors? What are other factors that could possibly affect cognitive computation? Previously, it has been known that working memory capacity, such as reading span, is highly interconnected with people’s processing of sentences. Would this working memory capacity affect language processing or scalar inferences among second language learners? More detailed discussion of working memory capacity and reading span task will be presented in the third experiment. Given that not many online experimental studies have been done to investigate scalar inferences among second language learners and the few existing studies still rely heavily on Truth Value Judgment Tasks or other offline methods, it is of great interest and importance to apply online techniques to elucidate scalar inferences and psychological reality among L2 language learners.

I now turn to the three experiments in this paper. In Experiment 1, I used the Truth Value Judgment Task and reaction times to examine the two interpretations of “some.” First, which theory, Default Inference or Relevance Theory, can be used to account for second language learners’ performances in scalar interpretation? How long do second language learners spend on processing scalar inferences? Both the offline Truth Value Judgment Task and the online reaction performances provide us a more in-depth window into the cognitive mechanism of scalar inferences among second language learners. If the Default Inference theory is correct, we should be able to observe more and faster acceptance of “some but not all” interpretation. In other words, there should be reliable differences in the acceptance of “some but not all” interpretation than in the acceptance of “some and possibly all”. On the contrary, if the Relevance Theory is correct, we would observe the opposite pattern in participants’ performances. That is, we should observe faster acceptance of “some and possibly all” interpretation. Experiment 2 used the same paradigm as Experiment 1 except that subjects were not given limitless time to respond. Instead, subjects were given a certain period of time to respond. This was done in order to further verify that people’s choice of interpretation would not change under the pressure of time. The second research question would thus be: Would scalar inferences remain the same under time pressure for second language learners? The predictions for the second experiment remain the same basically. If, under time constraint, comprehenders’ choose “some but not all” interpretation more and faster than “some and possibly all”, we have further evidence that Default Inference makes the right inference regarding comprehenders’ cognitive processing. On the contrary, if comprehenders choose “some and possibly all” more and faster than “some but not all”, we have evidence that the Relevance Theory is correct. Experiment 3 extended the findings from Experiment 1 and Experiment 2 and examined one important cognitive factor that contributes to human language processing: working memory capacity. Working memory capacity has been shown to affect sentence comprehension and students’ performance in scholarly aptitude tests. However, much less is known about the role of working memory capacity in second language research. In Experiment 3, I investigate this capacity during scalar inferences. Several research questions are of interest in this paper: The research question for the third experiment is: How would people with a high reading span differ from those with a low reading span? Do they show different patterns of scalar inference processing? We address these questions in the following three experiments.

Furthermore, since the current study aimed to use Mandarin learners of English as participants, two possible concerns should be addressed before running the following experiments. First of all, would Mandarin learners of English be able to read two interpretations of “some” in English? That is, would they be able to read the logical and pragmatic interpretations, just like English native speakers? To examine this issue, we conducted an experiment asking participants who did not participate in the following three experiments to evaluate both English and Mandarin stimuli sentences by using either “some but not all” interpretation or “some and possibly all” interpretation. For example, twenty participants were given instruction about using “some and possibly all” scenario to evaluate stimuli sentences based on 1–5 Likert Scale, with “1” meaning “very unlikely” and “5” meaning “very likely”. Another twenty participants were given instruction about using “some but not all” interpretation to evaluate stimuli sentences based on 1–5 Likert Scale. Then we compared whether there was a difference in their rating between these two interpretations. The average for “some but not all” interpretation was 4.38 while the average for “some and possibly all” interpretation was 4.12. Statistical analysis showed that there was also no difference in participants’ rating (*F*(1, 38) = 3.97, *p* > .05). In addition to this, we also checked whether there was a difference between English and Mandarin stimuli. Statistically, there was also no difference between English and Mandarin stimuli (*F*(1, 38) = 2.13, *p* > .05).

## Experiment 1

The first experiment set out to examine the first research question: Which theory, Default Inference or Relevance Theory, can be used to account for second language learners’ performances in scalar interpretation? Instead of forcing subjects to judge stimuli sentences either logically or pragmatically or to apply real world knowledge during the experiment, Mandarin learners of English were introduced to a context that depicted a condition that could support either a logical or a pragmatic interpretation. For example, a typical context could be: “John has many dictionaries. Some of the dictionaries are used.” In this context, it is possible to interpret *some* as either *some but not all* or *some and possibly all*. After reading the context, subjects were then presented with one of the following target sentences: “Some and possibly all of the dictionaries are used” or “Some but not all of the dictionaries are used.” A total of 40 sets of target sentences (see Appendix I) were used in the Experiment 1 with 20 of them using “some and possibly all” and 20 using “some but not all” in the questions. These target stimuli were then randomized with 40 control sentences (see Appendix II) so that subjects did not detect the manipulation of the experiment. Controls were designed not to include scalar inferences so that they could serve as the basis in the experiment. Typical control sentences would read, “Jennifer has four borrowed books. They were from the library.” The following target sentence could read, “Jennifer has four borrowed books from the library.”

After reading the target sentence, subjects need to respond by clicking either “True” or “False.” Two kinds of responses were recorded: subjects’ reaction times and their responses to the target sentence. Reaction times were measured as the time that subjects took to respond to the question as either true or false. If the Default Inference is correct, the percentage of true responses should be greater in “some but not all” interpretation than that in the “some and possibly all” interpretation. In addition, we can observe less time spent on interpreting *some* as *some but not all* since it is the default interpretation. On the other hand, percentage of false responses should be lower in “some but not all” interpretation than that in “some and possibly all” interpretation and the reaction time for false responses in the “some but not all” interpretation should be longer than that in the “some and possibly all” interpretation.

### Subjects and Procedures

Before conducting the experiment, the researcher has gained approval from Research Ethics Committee at National Taiwan University. The Chairperson of Research Ethics Committee was Shih-chung Hsieh. Thirty native Mandarin learners of English were recruited. They were all college or grad school students at National Taiwan University, ranging in age from 19 to 30. Among these participants, 16 were females while 14 were males. Prior to college, they all had at least eight years of learning English as a foreign language in elementary schools, junior high schools and senior high schools. In addition, these participants had passed either the intermediate level or high-intermediate level of the General English Proficiency Test (GEPT) held by The Language Training and Testing in Taiwan. The intermediate level of GEPT is equivalent to B1 level of Cambridge English Language Assessment and the high-intermediate level of GEPT is equivalent to B2 of Cambridge English Language Assessment. Passing the GEPT high-intermediate level has become one of the criteria at National Taiwan University before students graduate.

Subjects were greeted by the experimenter as they entered the laboratory. Before participating in the experiment, they were provided with consent forms, which detailed the rights of human subjects. Upon receiving their signatures, they were informed about the procedures of the current experiment. Once they had completed the experiment, subjects were offered NT$100 for their participation. Since the subjects in the current study were not native speakers of English, care was taken to ensure that all the words and the simple structures were familiar to them. Even though most of the vocabulary was not difficult, subjects needed to go through all the vocabulary used in the study (see Appendix III) and made sure that they understood all of the words before participating in the experiment. In addition, fifteen trials were presented to subjects to familiarize them with the experiment procedures. On the computer screen, subjects read stimuli like “John has many dictionaries. Some of the dictionaries are used.” The context lasted for 2500 milliseconds. After that the screen was replaced by a target sentence such as “Some but not all of John’s dictionaries are used” or “Some and possibly all of John’s dictionaries are used.” Subjects were prompted to make a true/false judgment by pressing the right/left shift key. Note that in Experiment 1, subjects can take as much time as possible to determine their responses. In addition to sentences containing scalar inferences, control sentences were also included in the experimental stimuli and they also required subjects to make true/false judgments. What is different is that there were correct responses for control sentences. Subjects who did poorly on the control sentences would be dropped for later analysis.

### Statistical Analysis and Results

**Fig 1:**
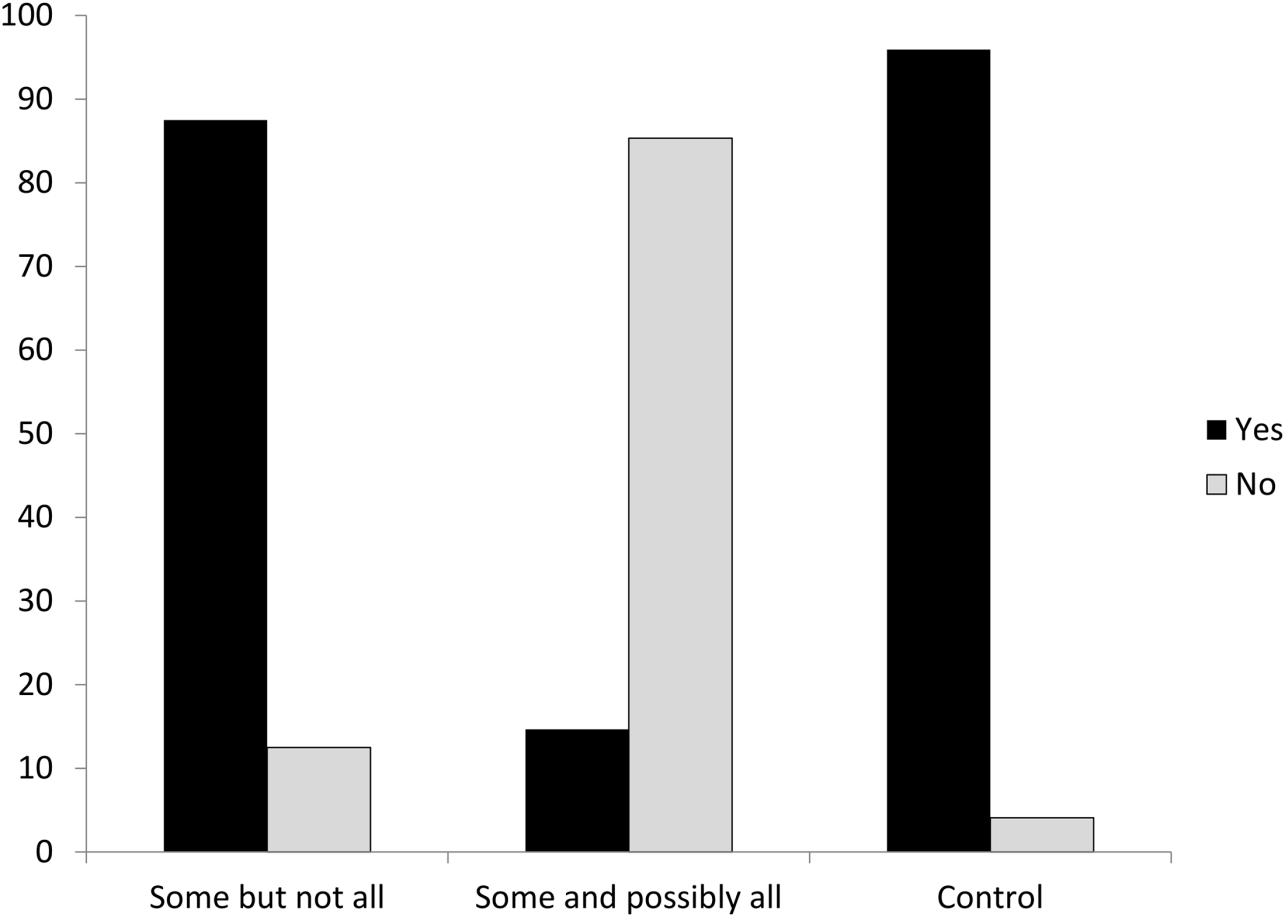
True/False Judgments in Experiment 1. When comprehenders were presented with “Some but not all” interpretation, they were more likely to respond “Yes” while when they were presented with “Some and possibly all” interpretation, they were more likely to respond “No.”

First of all, control sentence results showed that no subjects needed to be dropped for analysis since the percentage of correct response reached 92% for all subjects. Given that the dependent variable in the true/false judgment is not continuous, ANOVA analysis should not be applied in this context. Instead, categorical data analysis was performed. The results showed that both Chi-squared and Likelihood Ratio Chi-squared reached significance (*χ*^*2*^(2) = 1436.23, *p* < .00; Likelihood Ratio *χ*^*2*^(2) = 1413.68, *p* < .00). Furthermore, due to the binary features of the response variables, binary logistic regression was applied for further analysis. SAS output revealed that the convergence criterion had been satisfied. There was a main effect of the answer “yes” among three types of stimuli: *Some but not all*, *some and possibly all* and control sentences (Wald *χ*^*2*^(2) = 839.47, *p* < .00). Post-hoc multiple comparisons adjusted by Bonferroni correction revealed significant differences among three comparisons (Control vs *Some but not all*: Wald χ^*2*^(1) = 40.1392, p < .0001; *Control* vs *Some and possibly all*: Wald χ^*2*^(1) = 330.4069, p < .0001; *Some but not all* vs *Some and possibly all*: Wald χ^*2*^(1) = 65.2169, p < .0001). The statistical results showed that subjects’ acceptance of *some but not all* was significantly higher than their acceptance of *some and possibly all (as revealed in*: *Some but not all vs Some and possibly all*: *Wald χ*^*2*^*(1) = 65*.*2169*, *p <*.*0001)*. Similarly, subjects’ acceptance of the control sentences was reliably higher than their acceptance of *some and possibly all* (Wald *χ*^*2*^(1) = 330.4, *p* < .00). This is depicted in Fig 1.

The results of the true/false judgments showed that Mandarin second language learners of English displayed a much higher percentage of accepting *some but not all* than *some and possibly all* as the interpretation of “some.” When presented with *some and possibly all* as the interpretation of “some,” Mandarin learners of English tended to reject this interpretation more (85%) than accepting this interpretation (15%). A further discussion of this acceptance rate will be provided in the discussion section.

**Fig 2:**
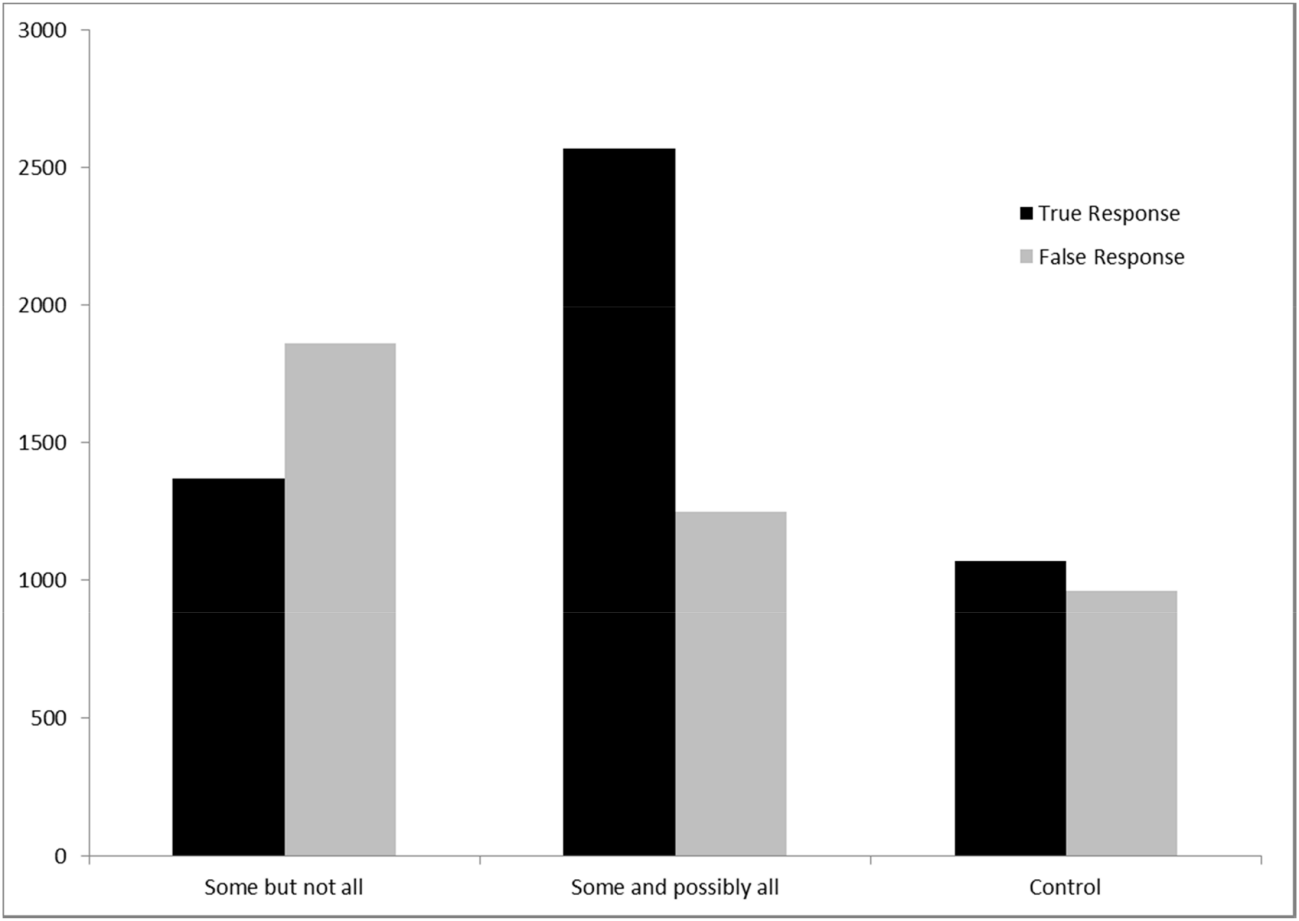
Reaction Times in Experiment 1. Comprehenders spend much less time in interpreting “some” as “some but not all” than as “some and possibly all”.

Fig 2 presents the reaction times data from the first experiment. Given that reaction time data belongs to gamma distribution, the reaction time data in this experiment was log-transformed so that they didn’t violate the basic assumptions of ANOVA. The results of analyses of true responses showed significant differences in reaction times among three types of conditions, i.e., *some but not all*, *some and possibly all* and *controls* (Subject analysis: *F*_*1*_(2, 87) = 166.7, *p* < .000; Item analysis: *F*_*2*_(2, 87) = 82.26, *p* < .000). In addition, omnibus post-hoc comparisons, adjusted by Bonferroni correction, revealed that there were significant differences in reaction times in each post-hoc comparison (all subject and item analyses: *p* < .01). Mandarin learners of English were faster in accepting *some* as *some but not all* than *some and possibly all* (both subject and item analyses: *p* < .000). Fig 2 shows that Mandarin learners, on average, only spent around 1350 milliseconds when interpreting *some* as *some but not all*. However, they spent almost twice as long (average reaction time: 2500 milliseconds) when responding to *some and possibly all*. Combined with the rejection rate in Fig 1, these results indicate that second language learners not only tended not to interpret *some* as *some and possibly all*, but also took much more time to ponder it. It is possible that they were trying to come up with a scenario that could fit a *some and possibly all* interpretation.

When comparing the control sentences with the logical and pragmatic interpretations of *some*, the results showed that even though subjects were faster in responding to *some but not all*, the reaction times were still significantly longer than those of the control sentences (both subject and item analyses: *p* < .000). The average time that people spent on responding to control sentences was around one second while they spent 1350 milliseconds on the *some but not all* condition. Note that control sentences are ordinary English sentences and they didn’t require subjects to make any inferences at all. The results suggest that Mandarin learners of English took around one second to judge these control sentences, much quicker than judging the two interpretations of *some*.

Fig 2 also demonstrates subjects’ reaction times to false responses. Again, log-transformation was also applied on reaction time data and they also revealed significant differences in reaction times among these three types of conditions: “some but not all”, “some and possibly all” and “control sentences” (Subject analysis: *F*_*1*_(2, 87) = 85.5, *p* < .000; Item analysis: *F*_*2*_(2, 87) = 62.15, *p* < .000). Bonferroni adjusted post-hoc comparisons also reveal reliable differences for each pair (all subject and item analyses: *p* < .05). Fig 2 shows that subjects spent much longer time on “some but not all” interpretation than on “some and possibly all” interpretation, indicating that it was harder for them to reject “some but not all” interpretation. What needs to be noted is that when rejecting “some and possibly all” interpretation, subjects spent roughly similar time as they spent in accepting “some but not all” interpretation.

### Interim Discussion

The goal of Experiment 1 was to investigate the cognitive mechanism of scalar inferences. To be more specific, Experiment 1 tried to determine which theory, Default Inference or Relevance Theory, was correct in predicting people’s derivation of scalar inferences. Default Theory predicts that people should spend less time on “some but not all” since it is the default scalar derivation and is thus less cognitively demanding. The Relevance Theory, on the contrary, predicts the opposite, suggesting that people should spend less time on “some and possibly all.” Combing results from both true responses and false responses, the results both showed that it was easier for Mandarin learners of English to accept “some but not all” interpretation. This could be revealed from the fact that they spent significantly less time when interpreting *some* as *some but not all* and more time on rejecting this interpretation. When the prompting sentence was, for example, “Some but not all of John’s books were used,” subjects took an average of 1350 milliseconds to respond. However, when the target sentence was “Some and possibly all of John’s books were used,” they took 2500 milliseconds on average to respond. Subjects spent almost twice as much time as they did in responding to *some but not all*, which could be used to indicate that they were trying to come up with a scenario to fit the situation. Note that Mandarin learners of English not only spent more time on “some and possibly all,” but also tended to reject this interpretation as well.

Even though this finding comes from Mandarin learners of English, it could still be used to support the Default Theory, rather than the Relevance Theory. Note that the results of Experiment 1 not only supported the Default Inference, but also were in line with the previous offline experimental results, which found that adults used more pragmatic inferences (i.e., *some but not all* interpretation) than children. Given that the second language subjects in the current study were also adults, the results thus not only confirmed previous findings but also offered further online experimental evidence on the time course of scalar inferences.

Finally, do scalar inferences take people more time to process? According to the results of Experiment 1, the answer seems to be positive. When scalar inferences such as *some* are involved, people usually take more time to process. The results can also be used to support the fact that cognitive resources are required for implicature calculation.

## Experiment 2

The results of Experiment 1 indicated that Mandarin learners of English prefer to interpret *some* as *some but not all*, as reflected by the results from both reaction time data and the Truth Value Judgment Task. However, since subjects were not required to make their responses within a certain period of time, it is arguably true that they might be able to come up with scenarios that were compatible with either *some but not all* or *some and possibly all*. Since the purpose of the experiment was *not* to let subjects brainstorm during experiments so that they could come up with a possible scenario to fit the interpretation, it was therefore necessary to know if subjects could still make the same responses when they were pressed for time. Experiment 2 set out to examine whether subjects interpreted *some* as *some but not all* or *some and possibly all* under the pressure of time. It was hypothesized that when people are under pressure to respond within a short range of time, they would not be able to brainstorm and possibly find a scenario to fit the other interpretation.

The results of the first experiment showed that people tended to spend around 2500 milliseconds when they were presented with *some* as *some and possibly all* and around 1300 milliseconds when presented with *some* as *some but not all*. It seemed that when presented with *some and possibly all*, subjects needed another 1200 milliseconds to evaluate the possibility of this interpretation of *some*. Experiment 2 thus gave subjects 1500 milliseconds to respond to the stimuli sentences. This was to make sure that subjects could still process the target sentence but couldn’t use real world knowledge or other techniques to come up with a possible scenario. Again, if the Default Inference is correct, the percentage of true responses, under time pressure, should still be greater in “some but not all” interpretation than that in the “some and possibly all” interpretation. In addition, we should still observe less time spent on interpreting “some” as “some but not all” since it is the default interpretation. On the other hand, percentage of false responses should be lower in “some but not all” interpretation than that in “some and possibly all” interpretation and the reaction time for false responses in the “some but not all” interpretation should be longer than that in the “some and possibly all” interpretation.

### Subjects and Procedures

Regarding the second experiment, ethics approval was gained from Research Ethics Committee at National Taiwan University as well. The Chairperson of Research Ethics Committee was Shih-chung Hsieh. Another thirty native Mandarin learners of English who did not participate in the first experiment were recruited. They were all college or grad school students at National Taiwan University, ranging in age from 18 to 25. Among these participants, 17 were females while 13 were males. Prior to college, they all had at least eight years of learning English as a foreign language in elementary schools, junior high schools and senior high schools. In addition, these participants had passed either the intermediate level or high-intermediate level of the General English Proficiency Test (GEPT) held by The Language Training and Testing in Taiwan. The intermediate level of GEPT is equivalent to B1 level of Cambridge English Language Assessment and the high-intermediate level of GEPT is equivalent to B2 of Cambridge English Language Assessment. Passing the GEPT high-intermediate level has become one of the criteria at National Taiwan University before students graduate.

The procedures used in Experiment 2 were the same as those used in the first experiment, except that subjects were told that they only had 1500 milliseconds to respond to the target sentence. Under the time constraint, if the Default Inference is correct in predicting the human scalar inferences, we should still be able to observe less time spent on *some but not all* interpretation since it is the default interpretation. If, on the contrary, the Relevance Theory is correct, we should observe the opposite pattern; that is, less time would be spent on choosing *some and possibly all* interpretation.

### Statistical Analysis and Results

**Fig 3:**
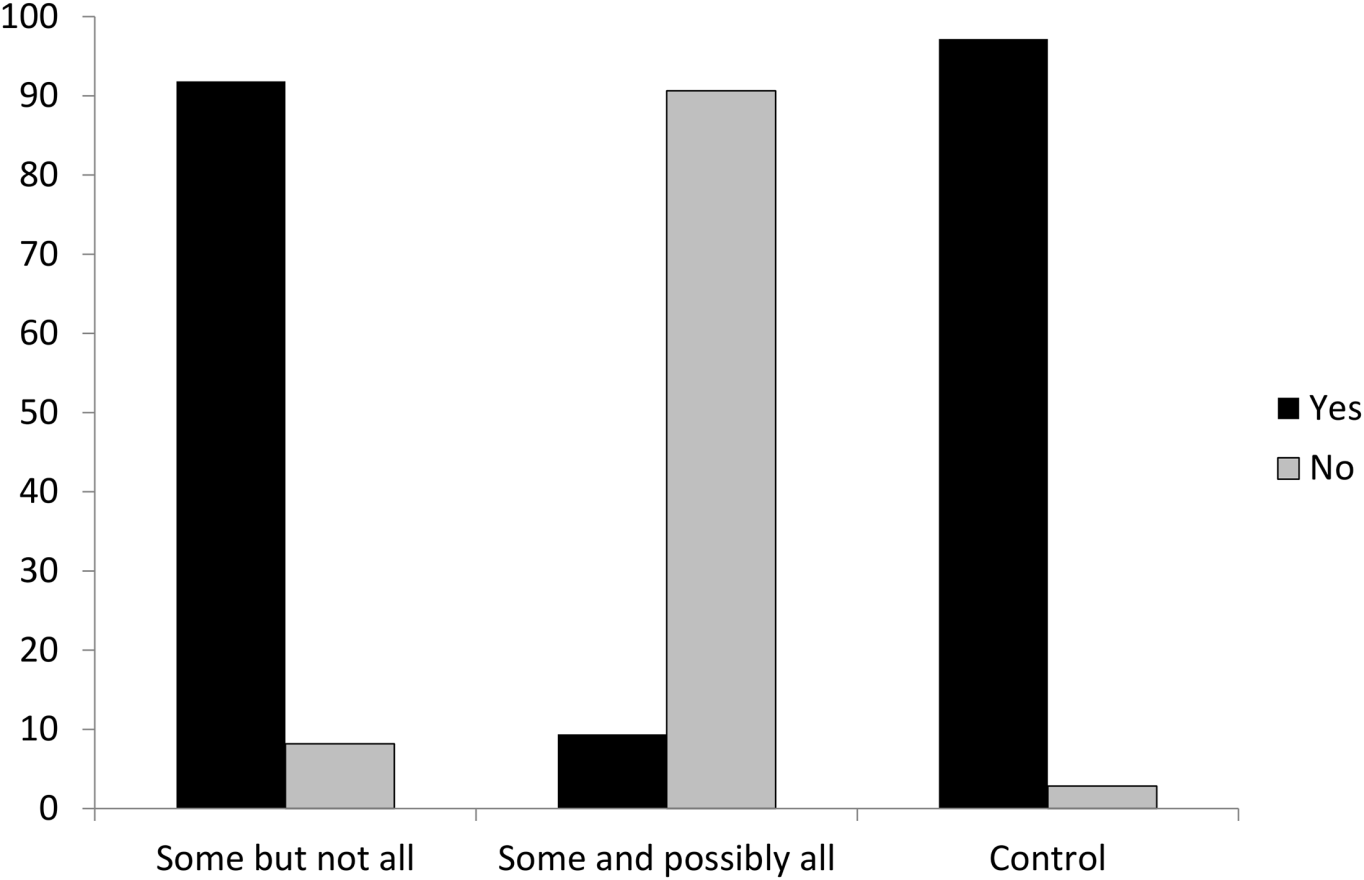
True/False Judgments in Experiment 2. When comprehenders were pressed for time, the percentage for them to accept “Some but not all” got higher than when they were not pressed for time.

The results from Experiment 2 showed that both Chi-squared and Likelihood Ratio Chi-squared reached significance (*χ*^*2*^(2) = 1729.6644, *p* < .00; Likelihood Ratio *χ*^*2*^(2) = 1720.98, *p* < .00). In addition, binary logistic regression was applied for data analysis. SAS output revealed that the convergence criterion was satisfied. There was a main effect of the answer “yes” among three types of stimuli: *some but not all*, *some and possibly all* and control sentences (Wald *χ*^*2*^(2) = 843.11, *p* < .00). Post-hoc multiple comparisons adjusted by Bonferroni correction revealed significant differences among three comparisons (Control vs Some but not all: Wald *χ*^*2*^(1) = 23.6861, *p* < .0001; Control vs Some and possibly all: Wald *χ*^*2*^(1) = 293.7725, *p* < .0001; Some but not all vs Some and possibly all: Wald *χ*^*2*^(1) = 92.0261, *p* < .0001). As in the first experiment, subjects’ acceptance of *some but not all* was significantly higher than their acceptance of *some and possibly all* (Wald *χ*^*2*^(1) = 92.02, *p* < .0001). By the same token, subjects’ acceptance of the control sentences was reliably higher than their acceptance of *some and possibly all* (Wald *χ*^*2*^(1) = 293.77, *p* < .0001), as indicated in Fig 3.

The results of the true/false judgments in Experiment 2 showed that when second language learners were pressed for time, the acceptance rate of interpreting *some* as *some but not all* remained the same as the results from Experiment 1. Nevertheless, the acceptance rate of interpreting *some* as *some and possibly all* dropped to less than 10 percent, as compared with nearly 20 percent in Experiment 1. It seemed that when Mandarin learners of English did not have enough time to think about the possibility of *some and possibly all*, they tended to reject this interpretation.

**Fig 4:**
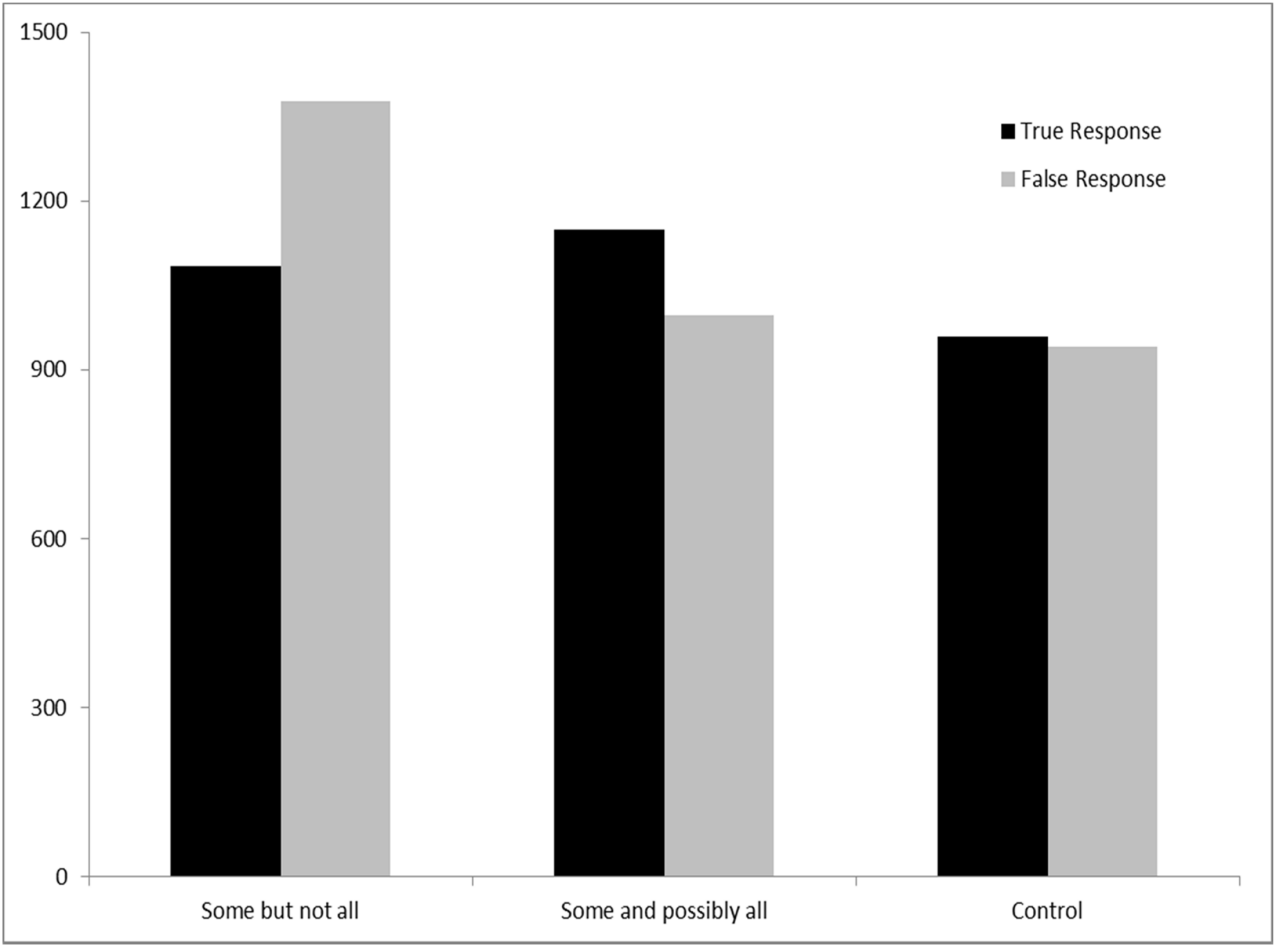
Reaction Times in Experiment 2. When comprehenders were pressed for time, they were still more likely and faster to choose “Some but not all” interpretation. Reading times for “Some but not all” were still a lot shorter than those for “Some and possibly all”.

Fig 4 presents the reaction times data for true and false responses from the second experiment. Given that reaction time data belongs to gamma distribution, the reaction time data in this experiment were also log-transformed so that they didn’t violate the basic assumptions of ANOVA. If we compare Figs 2 and 4, the first thing we would notice is that the reaction times for the *some and possibly all* condition have significantly dropped. In fact, the reaction times for all three types of sentences dropped considerably, possibly due to the time pressure. Still, regarding true responses, there was a significant difference in reaction times among the three types of interpretations (Subject analysis: *F*_*1*_(2, 87) = 5.44, *p* < .01; Item analysis: *F*_*2*_(2, 77) = 8.211, *p* < .01). Post-hoc analysis further revealed that there were reliable differences between *some but not all* and the *control* group (Subject analysis: *p* < .05; Item analyses: *p* < .01) and also between *some and possibly all* and the control group (Subject analysis: *p* < .00; Item analyses: *p* < .00). Nevertheless, there was no reliable difference in reaction times between *some but not all* and *some and possibly all* (Subject analysis: *p* = .063; Item analyses: *p* = .0576).

With respect to false responses, Fig 4 shows that subjects’ responses have also dropped dramatically. Log-transformed reaction time data revealed a reliable difference among the three types of interpretations (Subject analysis: *F*_*1*_(2, 87) = 3.18, *p* < .05; Item analysis: *F*_*2*_(2, 77) = 5.67, *p* < .05). Post-hoc analyses, adjusted by Bonferroni correction, further revealed that there were reliable differences among two groups (that is, between “some but not all” and “some and possibly all”: Subject analysis: *p* < .05; Item analyses: *p* < .05; between “some but not all” and control group: Subject analysis: *p* < .05; Item analysis: *p* < .05). However, there was no difference between “some and possibly all” and the control group (Subject analysis: *p* > .05; Item analyses: *p* > .05).

### Interim Discussion

The second experiment aimed to investigate whether Mandarin learners’ processing of scalar inferences would change if they were pressed for time due to the concern that subjects might be able to come up with a scenario to fit the alternative interpretation, i.e., *some and possibly all*. The results in Experiment 2 showed a huge drop in reaction times in subjects’ true responses when responding to *some and possibly all*. Statistics revealed that there were no differences in reaction times for true responses between *some but not all* and *some and possibly all*. However, reliable differences were shown in false responses. In addition, we observed an increase in rejection rate of *some and possibly all* conditions, revealing that when subjects were pressed for time and did not have sufficient cognitive resources, they were less likely to accept the *some and possibly all* interpretation. This evidence is in line with the results from Experiment 1 and serves as another piece of evidence that scalar processing still requires more cognitive capacity than simple structures. Finally, the results of Experiment 2 further supported the Default Inference account. This can be reflected by both the true/false judgment tasks and reaction times. Even though subjects were limited in time to make responses, their reaction times for *some and possibly all* were still longer than the reaction times for *some but not all*, even though the statistical analysis did not reach significant difference. Reliable differences, however, did show in the false responses, which lend further support for the interim conclusion. Interpreting *some* as *some and possibly all* thus seems to be more onerous than interpreting it as *some but not all*.

## Experiment 3

Experiment 2 showed that subjects’ derivation of scalar inferences were seriously influenced by the pressure of time. Since the time it takes to process language also correlates with cognitive capabilities, another interesting question may be posed: whether the capacity of cognitive system plays a role in scalar inferences. To be more specific, we want to know if there is a difference in people’s derivation of scalar inferences with regard to high versus low working memory capacity. Before delineating how to approach this research question, I will first briefly introduce working memory and its function with regard to language processing.

### Working Memory

Working memory [35–38] has long been one of the most important theoretical and central constructs in cognitive psychology due to its methodological advantages of measuring human cognitive capacities as well as its involvement in many complex human behaviors, such as comprehension, reasoning and problem solving. With working memory, we are able to store and retrieve information, and keep important information active and accessible under various circumstances. For instance, during a conversation, comprehenders need to get the lexical meaning of each word, build syntactic structures, and assign thematic roles. Unfortunately, not all these stages can be done simultaneously. Some of the initial items need to be held temporarily in the working memory so that they can be integrated with latter items of the string. Working memory plays a dominant role during this process since it involves temporary storage and manipulation of information that is assumed to be necessary for sentence comprehension. Baddeley and his colleagues [35–38] proposed a working memory model, which has captured the essence of how memory works during information processing. In its original model, working memory contained three major components: the central executive, the phonological loop and the visual-spatial sketchpad. In this model, the central executive functions like an attention-control center and the phonological loop is concerned with verbal and acoustic information processing. A fourth component, the episodic buffer, was added to the working memory model in 2000 [36]. The main function of the episodic buffer is to combine “information from different modalities into a single multi-faceted code” (p. 203).

Much effort has been devoted to measuring working memory capacity. So far, it has been found that human cognitive systems are sensitive to different working memory tests [39]. The most influential test of working memory capacity and sentence comprehension was designed by Daneman and Carpenter [40]. They developed a reading span task where subjects were required to read aloud a series of different sets of sentences starting with two sentences while maintaining the last word in their working memory. When they finished the target set, subjects were asked to recall the last words in correct order. If they succeeded in recalling the words, they were presented with increasingly longer sets of sentences. Their working memory capacity is reflected by the number of items they could recall correctly in serial order. Daneman and Carpenter have shown that the reading span task correlates well with subjects’ reading proficiency.

Studies on working memory and second language learning did not emerge until fairly recently [41–42], but whether working memory capacity can serve as a good predictor of second or foreign language learning is still not clear. One of the pioneers, Ellis, tried to use various kinds of span tasks to predict second language learning. He found that non-word repetition was the best predictor of second language learning. This finding was supported by other studies [42, 43, 44, 45] but not others [46]. The relationship between working memory capacity and scalar inferences among second language learners is much less cultivated in second language research. The experiment reported below aims to bridge this gap by examining whether individual differences in cognitive abilities affect the two interpretations of “some.” In addition, if they do, what kind of patterns would people with different working memory capacity show?

### Subjects and Procedures

The procedures used in Experiment 3 were the same as those used in Experiment 2, except that every subject needed to complete a reading span task before starting the scalar inference experiment. Again, the third experiment gained ethics approval from Research Ethics Committee at National Taiwan University. The Chairperson of Research Ethics Committee was Shih-chung Hsieh. Another thirty native Mandarin learners of English who did not participate in the previous two experiments were recruited. They were all college students at National Taiwan University, ranging in age from 19 to 23. Among these participants, 13 were females while 17 were males. Prior to college, they all had at least eight years of learning English as a foreign language in elementary schools, junior high schools and senior high schools. In addition, these participants had passed either the intermediate level or high-intermediate level of the General English Proficiency Test (GEPT) held by The Language Training and Testing in Taiwan. The intermediate level of GEPT is equivalent to B1 level of Cambridge English Language Assessment and the high-intermediate level of GEPT is equivalent to B2 of Cambridge English Language Assessment. Passing the GEPT high-intermediate level has become one of the criteria at National Taiwan University before students graduate.

The procedure of working memory span followed Daneman and Carpenter’s reading span task except that the sentences that the subjects read in this task were in Mandarin. The reason why Mandarin, instead of English, was used in the reading span task was that the reading span task was designed to probe into the cognitive capacity of participants and participants’ dominant language is usually the language that is used in this task since it wouldn’t interfere with the cognitive functions that researchers are interested. Using a second or foreign language, such as English, to probe into the cognitive capacity of participants would not lead to an accurate depiction of participants’ cognitive capacity since their performance would be influenced by many other confounding factors such as proficiency and syntactic structures of the foreign language. Subjects were tested individually in a quiet room and after the reading span task, they went on to participate in the scalar inference experiment. Following criterion from previous studies, subjects with reading span below 3 were labeled as low reading span group and those with reading span above 3 were labeled as high reading span group. Twenty native Mandarin students were recruited in Experiment 3. Among these 20 subjects, 8 of them were labeled as high reading span group while 12 of them were labeled as low reading span group.

Experiment three can be also examination of the two theories: the Default Inference and the Relevance Theory. Based on the results of the previous two experiments, we should still be able to observe more acceptance of *some but not all* interpretation from both high and low reading span groups. But how much time would each group spend remain to be examined. Do comprehenders from the high reading span group spend much less time in making decisions since they have more cognitive capacities? Or do they consider more possibilities regarding scalar inferences since their cognitive capacities allow them to do so?

### Statistical Analysis and Results

Most of the procedures used to analyze the true/false judgment data and reaction time data were like the procedures used in the previous two experiments, except that in Experiment 3, another between-group independent variable, i.e., reading span, was included in the current analysis. Again, Chi-squared analyses were performed for both high and low reading span subjects. In high reading span subjects, there were significant differences in responding “yes” to stimuli sentences (*χ*^*2*^(2) = 1284.99, *p* < .00; Likelihood Ratio *χ*^*2*^(2) = 1194.24, *p* < .00). In low reading span readers, there were also significant differences in producing “yes” to stimuli sentences (*χ*^*2*^(2) = 1300.02, *p* < .00; Likelihood Ratio *χ*^*2*^(2) = 1286.37, *p* < .00).

**Fig 5:**
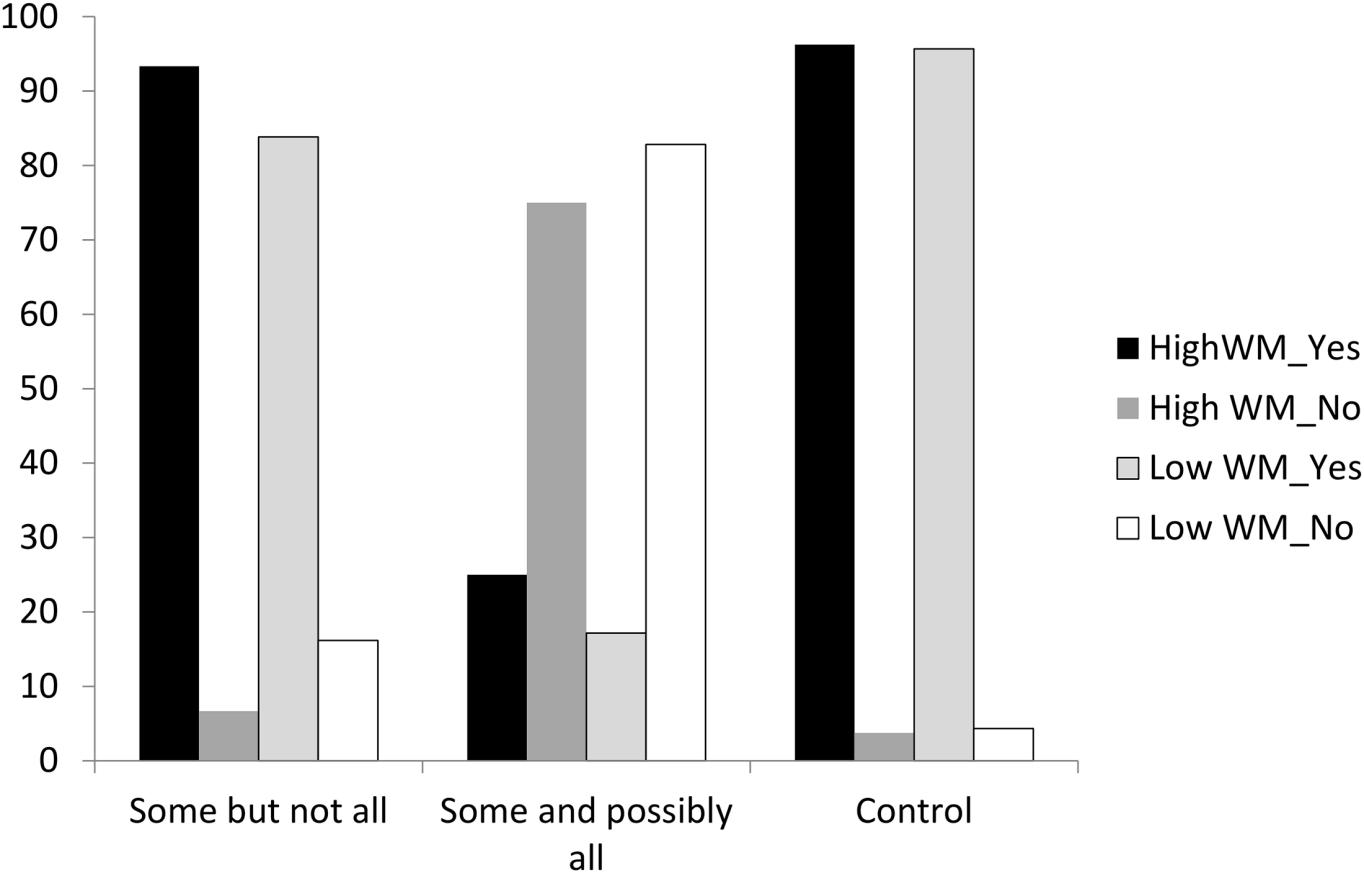
True/False Judgments b/w High and Low WM Capacity. Overall, comprehenders with both high and low working memory capacities were more likely to accept “Some but not all” interpretation than “Some and possibly all”. High-span comprehenders were also more likely to accept “Some but not all” than low-span comprehenders, as reflected by their higher percentage of acceptance rate.

Logistic regression was performed with predictor variables set as sentence types (i.e., “Some but not all,” “Some and possibly all” and “Control”) and reading span (i.e., high vs low reading spans). There were main effects for both sentence type (Wald *χ*^*2*^(2) = 1572.39, *p* < .00) and reading span (Wald *χ*^*2*^(1) = 29.28, *p* < .00). Post-hoc multiple comparisons adjusted by Bonferroni correction revealed significant differences among three comparisons (Control vs Some but not all: Wald *χ*^*2*^(1) = 66.1873, *p* < .0001; Control vs Some and possibly all: Wald *χ*^*2*^(1) = 563.6827, *p* < .0001; Some but not all vs Some and possibly all: Wald *χ*^*2*^(1) = 106.8035, *p* < .0001). It clearly indicated that high span readers produced significantly more “yes” responses than low span readers. In addition, subjects’ acceptance of “Some but not all” was reliably higher than their acceptance of “Some and possibly all”. There was also a reliable higher acceptance rate of “Control” than “Some and possibly all”. Last, a significant difference was found between “Some but not all” and “Control”. This is depicted in Fig 5.

**Fig 6:**
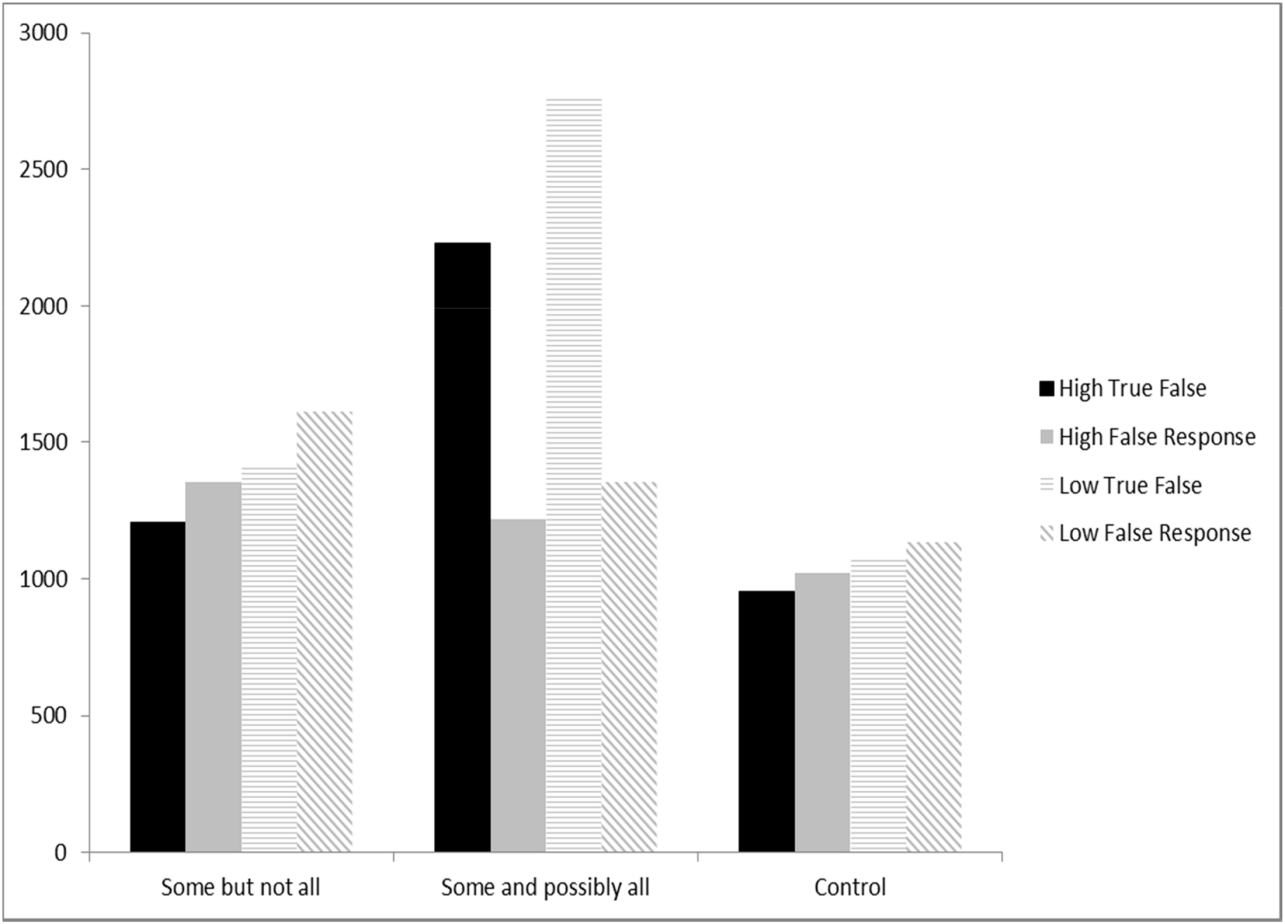
Reaction Times in Experiment 3. Comprehenders with both high and low working memory capacities spent much less time in accepting “Some but not all” interpretation. High-span comprehenders were also faster than low-span readers in accepting this interpretation.

Fig 6 presents the reaction time data for both true and false responses from the third experiment. All reaction time data were log-transformed before analyses. With respect to true responses, there were reliable reaction time differences among three stimuli sentences (Subject analysis: *F*_*1*_(2) = 142.84, p < .00; Item analysis: *F*_*2*_(2) = 200.46, p < .00). In post-hoc multiple comparisons, there were significant differences in all three pairwise comparisons (all subject and item analyses: p < .00). In addition, there was also a main effect in reading span (Subject analysis: *F*_*1*_(1) = 11.04, *p* < .01; Item analysis: *F*_*2*_(1) = 19.28, *p* < .00), which indicates that high span readers were faster in making responses during the experiment. In addition, there was no interaction effect (*p* > .05). It is also interesting to compare high versus low span readers’ performance under each sentence type category. In “Some but not all,” there was a marginally reliable difference in reaction times between high and low readers (*F*(1) = 3.457, *p* = .06). This suggests that even though both of the high and low reading span groups were fast in interpreting “some” as “some but not all,” the high reading span group was still a bit faster in making this interpretation. In “Some and possibly all,” high span readers were also much faster than low span readers (*F*(1) = 7.52, *p* < .01). However, in the “Control” group, the difference between the two groups was not significant (*F*(1) = 1.332, *p* = .253). The results of Experiment 3 suggest that high span readers were faster than low span readers in making judgments regarding “some but not all” and “some and possibly all.” The combined results from Figs 5 and 6 show that high span readers were faster in interpreting “some” as “some but not all.”

With respect to false responses, statistical analyses revealed two main effects (i.e., reading span and sentence stimuli type) but no interaction effect. There was a reliable difference in reading span difference (both Subject and Item analyses: *p* < .05). High span readers were faster in rejecting false responses than low span readers. Overall, for both groups of readers, they still took more time to reject “some but not all” interpretation than “some and possibly all” interpretation. There was also a reliable difference in sentence stimuli type (both Subject and Item analyses: *p* < .05). Post-hoc analysis, adjusted by Bonferroni correction, showed significant differences between two pairs of comparisons. When we compared high and low span readers’ performance for “some but not all” interpretation, we found a reliable difference in reaction times (*F*(1) = 3.07, *p* < .05), confirming our conclusion that high reading span group was still faster in making this interpretation. In “Some and possibly all” interpretation, high span readers were also much faster than low span readers (*F*(1) = 4.23, *p* < .05). However, in the “Control” group, the difference between the two groups was not significant (*F*(1) = 0.89, *p* > .05).

### Discussion

Experiment 3 aimed to examine whether cognitive capacity influenced Mandarin learners’ processing of scalar inferences. Previous studies have found that subjects with a high reading span were faster in processing sentences than subjects with a low reading span. In the current study, differences in reaction times and true/false judgments between high and low reading spans were also found. High reading span subjects were more likely and much faster in accepting a *some but not all* interpretation than low span readers, although both groups showed a very high acceptance rate of this condition.

What is more interesting and should be noted is that in *some and possibly all* conditions, high span second language learners were more likely to accept this interpretation than low span readers. The percentage for high span readers to accept *some and possibly all* was 25% while it was only 17% for low span readers. Even though the percentages do not seem to be quite different, their difference has reached significance, as indicated previously. A legitimate reason for such a high acceptance rate in this condition is that high span readers had more cognitive capacities to come up with possible scenarios to fit the *some and possibly all* condition. Both high and low reading span subjects showed longer reaction times in the *some and possibly all* condition. Despite this, high span readers still seemed to be a lot faster than low span readers. Finally, in the control sentences, both high and low span readers were very fast in accepting the sentences and there were no significant differences in their responses.

Overall, Experiment 3 found that working memory capacity did play a significant role in deriving scalar inferences among Mandarin learners of English. When people have more capacity in working memory, they are faster in evaluating sentences and at the same time, they are more likely to come up with possibilities to fit the scenario.

## Overall Discussion

The three experiments conducted in the current study tried to investigate scalar inferences among Mandarin learners of English by using online experiments. To be more specific, the current three experiments tried to examine which of the two theories, Default Inference or Relevance Theory, is correct in predicting Mandarin learners’ processing of scalar inferences. According to Default Inference, it predicts that interpreting “some” as “some but not all” is the default interpretation that learners will come up with and thus it will be cognitively less onerous for interlocutors. On the contrary, Relevance Theory predicts that the listener would tend to go with the logical interpretation, thus interpreting “some” as “some and possibly all.” If there is a need for the listener to go further, s/he would adopt the “some but not all” pragmatic interpretation.

So far, most of the studies on scalar inferences have focused on native speakers’ offline judgments of scalar inferences or on children’s interpretations of sentences containing scalars. None of them have used online measures to examine second language learners’ processing of scalars. Considerable disagreement has arisen out of these offline studies. The current study thus aims to bridge this gap by using reaction times and true/false judgment tasks to investigate this issue.

Across the three experiments, it has been found that Mandarin learners’ acceptance rate of *some but not all* was higher than their acceptance rate of *some and possibly all*. This evidence was also backed up by faster reaction times that subjects demonstrated to *some but not all*. It indicates that subjects did not hesitate to interpret *some* as *some but not all*. The overall reaction time for *some but not all* was about half as fast as that for *some and possibly all*. Another interesting issue that can be observed in the current study is that scalar inferences did consume more time than simple structures. Results showed that even though subjects’ reaction times in *some but not all* were faster than *some and possibly all*, they were both slower than the reaction times in *control* stimuli.

Faster reaction times and higher acceptance rate of *some but not all* could be used as a piece of evidence to support the Default Inference, which hypothesized faster responses for the *some but not all* interpretation. Given that the reaction times in *some but not all* were faster than *some and possibly all* and were more similar to the reaction times for control stimuli, the evidence can be used to indicate that generating a *some but not all* interpretation was less effortful and could be the default interpretation. In addition, the extra reaction times in *some and possibly all* conditions can be used to explain that when the default interpretation is not correct, people take time to reject such an interpretation.

Further evidence in support of a Default Inference account comes from the results of Experiment 2, which limited subjects’ responses to only 1500 milliseconds. In this situation, subjects’ rejection rate of *some and possibly all* was higher than the rejection rate in Experiment 1. This indicates that when people do not have enough time to try to come up with a suitable scenario to fit *some and possibly all*, they tend to reject it.

Besides finding compelling evidence in support of the Default Inference account, this paper also addressed the role of working memory capacity in scalar inferences. The results in Experiment 3 showed that learners with a high reading span are more likely to process scalar inferences faster and also more likely to generate possible scenarios to fit *some and possibly all*, as indicated by its higher acceptance rate. However, for subjects with a low reading span, they were slower and possibly more prone to making pragmatic mistakes. Taken all together, the three experiments in this paper demonstrate that experimental procedures help tease apart competing theories raised and contribute to our knowledge of current pragmatic theories.

## Appendix I: Experimental Stimuli

1. Mary had many sisters. Some of the sisters were married.

2. Peter owns many keys. Some of the keys are lost.

3. Dennis had a lot of pencils. Some of the pencils were used.

4. May has a lot of flowers. Some of the flowers are yellow.

5. Daniel had many books. Some of the books were dirty.

6. Martha has many brothers. Some of her brothers are really tall.

7. Jessica got a lot of assignments. Some of her assignments were difficult.

8. The movie star has many fans. Some of the fans were teenagers.

9. The teacher has many students. Some of the students are diligent.

10. The man had many uncles. Some of his uncles were rich.

11. The assistant mailed many letters. Some of the letters were long.

12. The manager has many employees. Some of the employees were old.

13. The clerk has got many complaints. Some of the complaints are about his attitude.

14. The cowboy has many horses. Some of the horses are sick.

15. The book has many authors. Some of the authors are female.

16. The computer has found many viruses. Some of the viruses are removable.

17. This city has many old buildings. Some of the old buildings are collapsing.

18. There are many movies on this website. Some of the movies are classic.

19. There are many people in that house. Some of the people are really old.

20. That tall building has many windows. Some of the windows are broken.

21. The patient has got many children. Some of the children are still young.

22. There are many girls in this club. Some of the girls are good-looking.

23. The apartment has many rooms. Some of the rooms are small.

24. There are many photos in that book. Some of the photos are beautiful.

25. The old man has many dogs. Some of the dogs are cute.

26. The store had many customers. Some of the customers were rude.

27. This campus has many trees. Some of the trees are tall.

28. Tom has many gardeners. Some of the gardeners are from the south.

29. This company has many managers. Some of the managers have just retired.

30. That old doctor has a lot of patients. Some of the patients hate him.

31. The farmer has grown a lot of apples. Some of the apples are green.

32. The student has many notebooks. Some of the notebooks are dirty.

33. The king has a lot of servants. Some of the servants are proud.

34. Jasmine went to many different countries. Some of the countries were beautiful.

35. Nicole has bought many dolls. Some of her dolls are really delicate.

36. Johnny prepared many dishes. Some of the dishes tasted great.

37. The policeman has caught many bad guys. Some of the bad guys are really evil.

38. The little boy has many pets. Some of his pets are dogs.

39. Tom’s parents have many bought apartments. Some of the apartments are huge.

40. The professor is working on many projects. Some of the projects are complicated.

## Appendix II: Control Sentences

1. Natalie has got many rejections. She is very sad.

2. The old painter lost his salary again. His wife is mad.

3. The businessman loves to travel. He has been to many different countries.

4. Mr. Johnson fired one of his employees. The employee stole his money.

5. The manager is mad. One of his workers got into a fight.

6. The student did not finish his report. He failed the class.

7. The mayor is sick. He needs to see a doctor.

8. Tim doesn’t like his teacher. He thinks she is too strict.

9. Carolyn loves sports. She plans to join the school team.

10. Amanda wants to study abroad. She is trying to earn more money.

11. The king has many knights. They are all very brave.

12. That single mom has two sons. They both go to high school.

13. Kate likes horror movies. She enjoys watching them at night.

14. Amy’s mom needs help. She has problems cleaning a big house.

15. The vending machine has many different products. All of them are cheap.

16. Nurses in that hospital are very busy. They need to take care of many patients.

17. A police officer is talking to a young man. He might have stolen something.

18. Richard has got many presents for his birthday. One of them is a toy car.

19. Many students find this course difficult. They can’t figure out the difficult content.

20. Martha wants to have a baby sister. But her mom wants a baby boy.

21. Many of the queen’s maids are young. They love to work in the palace.

22. John can’t wait to see his father, who has been away for one year.

23. Nancy’s mother went to see a doctor. She has been ill for a long time.

24. Vivian is lucky. She married her high school sweet heart.

25. The driver got a ticket because he went through a red light.

26. The store is not open today because the clerk overslept.

27. The babysitter is playing with the kid, who is only four years old.

28. The salesman is good at selling his products. He made lots of money.

29. The swimmer didn’t see the sign which says, “No Swimming.”

30. The singer has a lot of fans. She constantly meets with them to have fun.

31. The girl wants to be a popular singer but she doesn’t have a beautiful voice.

32. The actor is memorizing his lines, which are quite hard.

33. Mandy loved an expensive watch. She finally asked her husband to buy it for her.

34. The concert has attracted many people. All of them love classical music.

35. Brandon’s grandfather fought in the war. He is proud of him.

36. The writer has to take a part-time job because his novel didn’t sell well.

37. Diane’s parents are rich. They paid to let her go to the best school.

38. The party was fun. Many people didn’t want to leave.

39. Both of her children like to play basketball. They are professional players now.

40. Ron has been studying very hard for the exams. He finally got excellent grades.

## Appendix III: Vocabulary Used in the Experiment

Instruction: Below is a list of the vocabulary used in the experiment. Please read all of them and check if there are any words you don’t understand. If there are any words you don’t understand, feel free to ask the experimenter for their meanings.

1. sisters

2. married

3. keys

4. old

5. pencils

6. flowers

7. yellow

8. books

9. tall

10. used

11. brothers

12. assignments

13. difficult

14. movie star

15. fans

16. teenagers

17. hardworking

18. uncles

19. rich

20. assistants

21. letters

22. lost

23. employees

24. manager

25. clerk

26. complaints

27. cowboy

28. sick

29. horses

30. authors

31. female

32. computers

33. viruses

34. removable

35. buildings

36. collapsing

37. movies

38. classic

39. citizens

40. building

41. broken

42. window

43. patient

44. single

45. club

46. children

47. apartment

48. room

49. photos

50. beautiful

51. huge

52. customers

53. rude

54. gardeners

55. Tainan

56. retire

57. farmer

58. notebooks

59. servant

60. proud

61. country

62. beautiful

63. dolls

64. delicate

65. policeman

66. dish

67. evil

68. apartments

69. pets

70. dogs

71. professors

72. projects

73. complicated

74. owns

75. cute

76. rejections

77. painter

78. salary

79. mad

80. businessman

81. travel

82. different

83. countries

84. employees

85. fight

86. finish

87. report

88. fail

89. mayor

90. strict

91. abroad

92. earn

93. knights

94. brave

95. single

96. vending machine

97. products

98. cheap

99. police officer

100. content

101. maids

102. stole

103. palace

104. clerk

105. overslept

106. swimmer

107. fans

108. memorize

109. expensive

110. husband

111. concert

112. attract

113. classical

114. fought

115. professional
